# Inhibition of the *norA* gene expression and the NorA efflux pump by the tannic acid

**DOI:** 10.1038/s41598-023-43038-5

**Published:** 2023-10-13

**Authors:** Saulo Relison Tintino, Polrat Wilairatana, Veruska Cintia Alexandrino de Souza, Julia Mariana Assis da Silva, Pedro Silvino Pereira, Cícera Datiane de Morais Oliveira-Tintino, Yedda Maria Lobo Soares de Matos, João Tavares Calixto Júnior, Valdir de Queiroz Balbino, José P. Siqueira-Junior, Irwin Rose Alencar Menezes, Abolghasem Siyadatpanah, Henrique Douglas Melo Coutinho, Tereza Cristina Leal Balbino

**Affiliations:** 1 Laboratory of Microbiology and Molecular Biology (LMBM), Department of Biological Chemistry/CCBS/URCA, Recife, Brazil; 2 Department of Clinical Tropical Medicine, Faculty of Tropical Medicine, Mahidol University, Bangkok, 10400 Thailand; 3 Department of Microbiology, Aggeu Magalhães Institute, Oswaldo Cruz Foundation (Fiocruz), Recife, Brazil; 4 Department of Genetics, Federal University of Pernambuco, Recife, Brazil; 5 Laboratory of Microrganism Genetics (LGM), Department of Molecular Biology/CCEN/UFPB, Recife, Brazil; 6 Infectious Diseases Research Center, Gonabad University of Medical Science, Gonabad, Iran

**Keywords:** Antimicrobial resistance, Membrane proteins

## Abstract

The NorA efflux pump of *Staphylococcus aureus* is known to play a major role in the development of resistance against quinolone drugs by reducing their concentration inside target pathogens. The objective of this study was to evaluate the ability of tannic acid to inhibit the gene expression of the NorA efflux pump in *Staphylococcus aureus* and to evaluate the in silico effect on the pump. Efflux pump inhibition was evaluated by fluorimetry. The checkerboard method evaluates the effect of the test substance in combination with an antimicrobial at different concentrations. To gene expression evaluation NorA the assay was performed using: a sub-inhibitory concentration preparation (MIC/4) of the antibiotic; a sub-inhibitory concentration preparation (MIC/4) of the antibiotic associated with tannic acid at a sub-inhibitory concentration (MIC/4). In this study, docking simulations were performed by the SWISSDOCK webserver. The ability of tannic acid to inhibit the NorA efflux pump can be related to both the ability to inhibit the gene expression of this protein, acting on signaling pathways involving the ArlRS membrane sensor. As well as acting directly through direct interaction with the NorA protein, as seen in the approach and in silico and in vitro per checkerboard method and fluorimetry of bromide accumulated in the cell.

## Introduction

*Staphylococcus aureus* is the leading cause of human infectious diseases worldwide, ranging from superficial skin lesions to systemic and life-threatening infections such as osteomyelitis, endocarditis, pneumonia and septicemia. *S. aureus* virulence has been associated with the production of a large number of extracellular toxins, enzymes and cell-surface-associated proteins encoded by diverse genes^1^.

The *S. aureus* NorA efflux pump, a member of the major facilitator superfamily (MFS), is known to play a major role in the development of resistance against quinolone drugs by reducing their concentration inside target pathogens. NorA is also a proton motive force which mediates the intrinsic resistance of many unrelated drugs, including the monocationic compound ethidium and the hydrophilic fluoroquinolone ciprofloxacin^2^.

The NorA efflux pump present in *S. aureus* is expressed by the *norA* gene located in the Smal D fragment of the *S. aureus* chromosome^3^. The NorA efflux pump is characterized by the presence of 12 transmembrane segments^3,4^ and is related to the Bmr efflux pump present in *Bacillus subtilis*^5^. The NorA protein is predicted to have 388 amino acids with a molecular mass of 42,385^6^.

The expression of MDR efflux pumps is usually tightly regulated by transcriptional regulators acting at different levels. Efflux pump genes are usually expressed at low levels, at least in the laboratory setting, with physiological high-level expression requiring the presence of an effector. Notably, antibiotics are not among the best efflux pump expression inducers, which suggests the extrusion of antibiotics currently in use for the treatment of infectious diseases is not always the pump’s original function^7,8^. Non-physiological, inherited high-level efflux pump expression is achieved through mutations in elements involved in their regulation. These mutants can be selected under antibiotic therapy, hence contributing to resistance acquisition by microbial pathogens during treatment^9^.

Although efflux pump expression is downregulated in the absence of effectors triggering their expression^10^, the strength of this regulation is not the same for all efflux pumps. Each bacterial species contain a few efflux pumps which are constantly expressed (although at a low level) and which contribute to intrinsic resistance^11^, while other efflux pumps do not contribute to intrinsic resistance as their expression is tightly downregulated.

Knowledge of which efflux pumps contribute to the intrinsic resistance of bacterial pathogens is of great interest for the development of specific inhibitors against such pumps as these inhibitors will increase the susceptibility of mutants overexpressing these pumps to antibiotics, as well as increase the activity of antibiotics against wild-type strains expressing physiological levels of the aforementioned efflux pumps^12^.

The identification of plants capable of inhibiting efflux pumps is important as they provide a potential for lead optimization and future use with an existing antibacterial previously rendered ineffective due to MDR pumps in both Gram-positive and Gram-negative bacteria^13^. Several strategies for inhibiting efflux pumps exist, among which those involving efflux pump genetic expression inhibition have been highlighted as a new alternative. Plant-based products have stood out for their ability to inhibit efflux pumps, however their ability to inhibit NorA expression is still poorly understood^14^.

Several plants containing tannins in their composition are used in natural medicine against bacterial infections^15^. Tannins are widely distributed throughout the plant kingdom with tannic acid being a principal tannin. The term tannin is ordinarily used as a synonym for tannic acid. Tannic acid has numerous industrial, pharmacological and food additive applications. Tannic acid is reported to be toxic to animals if injected into the bloodstream or consumed in large quantities. Tannic acid is also a potent antioxidant and has exhibited anti-mutagenic, anti-carcinogenic and antibacterial activity^16,17^.

Tannic acid is one of the main examples of tannins which can be efficiently extracted from natural sources with high efficienc. Besides haver studies that prove its anticancer and antioxidant activity^19,20^. Point out that because tannic acid has affinity protein strong, it can act directly about NorA, or about signaling proteins necessary for NorA to activation^21^. The merit of this study largely consists to complement others previously published study that shows direct inhibition in the flow pump by reducing the minimum inhibitory concentration^22^. Reinforcing the potential of this compound as efflux pump inhibitor.

Therefore, the objective of this study was to evaluate the ability of tannic acid to inhibit NorA efflux pump gene expression in *Staphylococcus aureus*, as well as to evaluate the in silico effect of the tannic acid interaction with the NorA pump.

## Materials and methods

### Bacterial strains

The *S. aureus* SA-1199B strain, which overexpresses the *norA* gene encoding the NorA efflux protein responsible for extruding hydrophilic fluoroquinolones and other drugs such as DNA-intercalating dyes, was used. The strain, kindly provided by Prof. S. Gibbons (University of London), were maintained in blood agar base (Laboratorios Difco Ltda., Brazil) slants and, prior to use, the cells were grown overnight at 37 °C in Heart Infusion Agar slants (HIA, Difco). The *S. aureus* SA-1199 strain wild strain with low level of NorA expression and *S. aureus* ATCC 25,923 that does not have the NorA efflux pump.

### Drugs

The tannic acid and norfloxacin were obtained from Sigma Aldrich Co. Ltd., both of which were initially dissolved in dimethyl sulfoxide (DMSO) and afterwards in distilled, sterile water until reaching a concentration of 1024 μg/mL. The solutions were stored at − 20 °C and kept protected from the light. The carbonyl cyanide m-chlorophenylhydrazone (CCCP), ethidium bromide were obtained from Sigma Aldrich Co. Ltd.

### Sample preparations for real time PCR assays

The samples were prepared using the minimum inhibitory concentration (MIC) as obtained in Tintino et al.^18^ The assay was performed using: a sub-inhibitory concentration preparation (MIC/4) of the antibiotic; a sub-inhibitory concentration preparation (MIC/4) of the antibiotic associated with tannic acid at a sub-inhibitory concentration (MIC/4); and a growth control, i.e. the microorganism alone. The preparations were cultured in BHI (Brain Heart Infusion) culture medium for 24 h at 37 °C and then centrifuged at 3000 rpm for pellet formation, procedures performed prior to RNA extraction.

### Determination of Ethidium bromide fluorimetry in Gen5™ microplate reader

It was determined by the accumulation of bromide in the bacterial inside, with the strain SA1199B, an inoculum was made to revive the strains for 24 h at 37 °C and the inoculum was prepared in Mcfarland (0.5). Subsequently, a 96-well plate was prepared with solutions containing: 1—Ethidium bromide (MIC/4) + Bacteria + Culture medium (BHI 1%) + Tannic acid (MIC/4); 2—Ethidium bromide + Bacteria + Culture medium (BHI 1%) + Tannic acid (MIC/4); 3—Tannic acid (MIC/4) + Bacteria + Culture medium (BHI1%); 4—Bacteria + 5 Culture medium (BHI 1%). After preparation, the plate was incubated for 6 h in a bacteriological oven, then centrifuged at 3000 rpm, followed by washing the medium and resuspended in saline solution. The fluorimetry reading was performed using a Gen5™ Microplate Reader type plate reader, Software for Windows, of BioTek^®^ Instruments, Inc, with a reading in the range of 530 nm excitation and 590 nm emission.

### Determination of ethidium bromide fluorimetry in UV transilluminator

For plate analysis were used: strain 1199, wild with respect to 1199B with low NorA gene expression (right side), and a reference strain without the gene expression of *Staphylococcus aureus* pumps ATCC 25,923 (left side), sown on HIA (Heart Infusion Agar) for 24 h at 37 °C. Petri dishes were prepared in the following situations: 1—Carbonylcyanide m-chlorophenyl-hydrazone (CCCP-MIC/4) + bacteria + HIA culture medium + Ethidium bromide; 2—bacteria + culture medium (HIA) + Ethidium bromide; 3—Tannic acid + bacteria + culture medium (HIA) + Ethidium bromide. Being incubated for 24 to 37 °C C, with visual reading in UV Transilluminator.

### Gene expression evaluation

RNA extraction from the *S. aureus* strain was performed using the SV Total RNA Isolation System (Promega, USA) according to the manufacturer’s instructions. Complementary DNA (cDNA) was synthesized from total RNA by reverse transcription (RT) using the GoScript Transcription System (Promega, USA). NorA expression was assessed by qPCR, performed in triplicates using a Power SYBR^®^ Green PCR Master Mix (Applied Biosystems Co., UK) and an ABI PRISM 7500 sequence detector (Applied Biosystems, Foster City, CA). cDNA was used as a template in 20 μl reaction, including 10 μl Power SYBR^®^ Green and 1 pmol of each primer. The 16S gene was used as an endogenous control. The primers used in this study are described in Table 1. The relative *norA* gene expression was determined using the ΔΔC_T_ method.

**Table 1:** Primers used in this study.

Gene	Primers	Sequence (5′–3′)	Size (bp)	References
*norA*	*norA*-Fw	5′-TTCACCAAGCCATCAAAAAG-3′	620	Couto et al. (2008)
	*norA*-Rv	5′-CTTGCCTTTCTCCAGCAATA-3′		
16S	16S-Fw	5′-GTAGGTGGCAAGCGTTATGCC-3′	228	Lucero et al. (2013)
	16S-Rv	5′-CGCACATCAGCGTCAG-3′		

### Checkerboard

**Figure 1:**
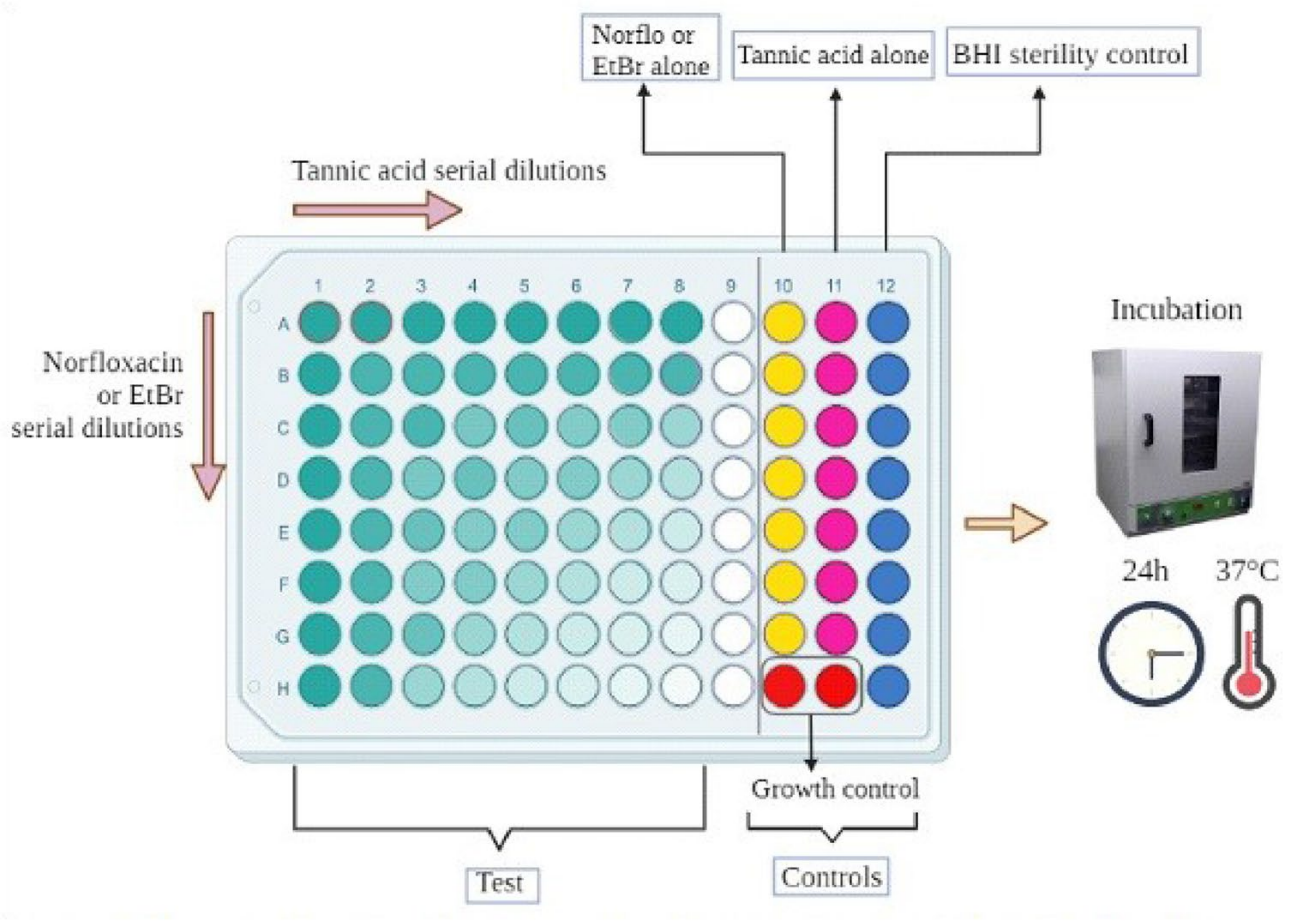
Representation of the microbiological checkerboard method. Source: The authors.

The checkerboard method evaluates the effect of the test substance in combination with an antimicrobial at different concentrations. It is a broth microdilution method similar to that used for standard MIC determinations. In a 96-well microtiter plate, the inoculum was distributed in 10% BHI, with 64 wells destined for the test, representing an 8 × 8 area of the plate; one 8-well column for the MIC of tannic acid, another column of wells for the MIC of norfloxacin or ethidium bromide. Other areas of the plate were reserved for inoculum growth and medium sterility controls. Then, in the 8 × 8 area of the test, microdilution was carried out horizontally with tannic acid and then vertically with the antibiotic, both at concentrations ranging from 512–4 µg/ml. In another plate, the same procedure was performed using ethidium bromide instead of norfloxacin (Fig. 1). The assay was performed in triplicate: three plates simulating the same test conditions. The plate was incubated for 24 h at 37 °C. Finally, resazurin was applied as a growth indicator, and the MIC of each substance alone and the MIC of compounds in combination were read. To analyze the effect obtained, it is necessary to obtain the value of the Fractional inhibitory concentration index (FICI). For this, the values obtained were applied to the following formula to obtain the fractional inhibitory concentration (FIC):

$${\text{FIC}}\;{\text{of}}\;{\text{compound}}\;{\text{A}}\;{\text{(FIC}}_{{\text{A}}} {)} = \frac{{{\text{MIC}}\;{\text{of}}\;{\text{compound}}\;{\text{A}}\;{\text{in}}\;{\text{combination}}}}{{{\text{MIC}}\;{\text{of}}\;{\text{compound}}\;{\text{A}}\;{\text{alone}}}}$$

$${\text{FIC}}\;{\text{of}}\;{\text{compound}}\;{\text{B}}\;{\text{(FIC}}_{{\text{B}}} {)} = \frac{{{\text{MIC}}\;{\text{of}}\;{\text{compound}}\;{\text{B}}\;{\text{in}}\;{\text{combination}}}}{{{\text{MIC}}\;{\text{of}}\;{\text{compound}}\;{\text{B}}\;{\text{alone}}}}$$

The sum of FICs indices of two compounds in the combination was calculated as follows: FIC_A_ + FIC_B_ = FICI, or FIC index. The interaction is synergistic when the FICI value was ≤ 0.5, additive if the FICI was > 0.5 and ≤ 1.0, indifferent if the FICI index was > 1.0 and ≤ 2.0, and antagonistic if the FIC index was greater than 2.0.

### Molecular docking procedure

In this study, docking simulations were performed by the SWISSDOCK webserver (www.swissdock.ch/)^21^. The 3D tannic acid coordinates were generated using the CORINA online server. Molecular Networks. CORINA online Demo. Available online at: http://www.molecularnetworks.com/online_demos/corina_demo (accessed on the 27th of February 2013).

Prior to performing molecular docking, the NorA protein was modeled by Dos Santos et al.^22^ and all files were prepared using the Dock prep tool available in the free UCSF chimera software package. Docking runs were performed blind by covering the entire protein and defining a region of interest with protein coordinates (x = −29.78, y = 49.65, z = 71.78 and box size with x = 46.00, y = 38.00 and z = 30.00) as binding pocket to ensure blind docking approach. The docking results were viewed with the help of the UCSF Chimera visualization program and the Discovery studio (DS) was utilized to detail how tannic acid interacted with NorA.

### Statistical analysis

The relative expression of the *norA* gene was determined using the ΔΔC method. Gene expression assays were performed in triplicate. Statistical analysis was performed by Kruskal–Wallis, followed by Dunn post-hoc test. The checkerboard and fluorescence analysis was performed by the geometric mean of the results, carried out through the GraphPad Prism 5.0 software, applying two-way ANOVA, and the post hoc Bonferroni.

## Results

### NorA quantitative real-time PCR

**Figure 2:**
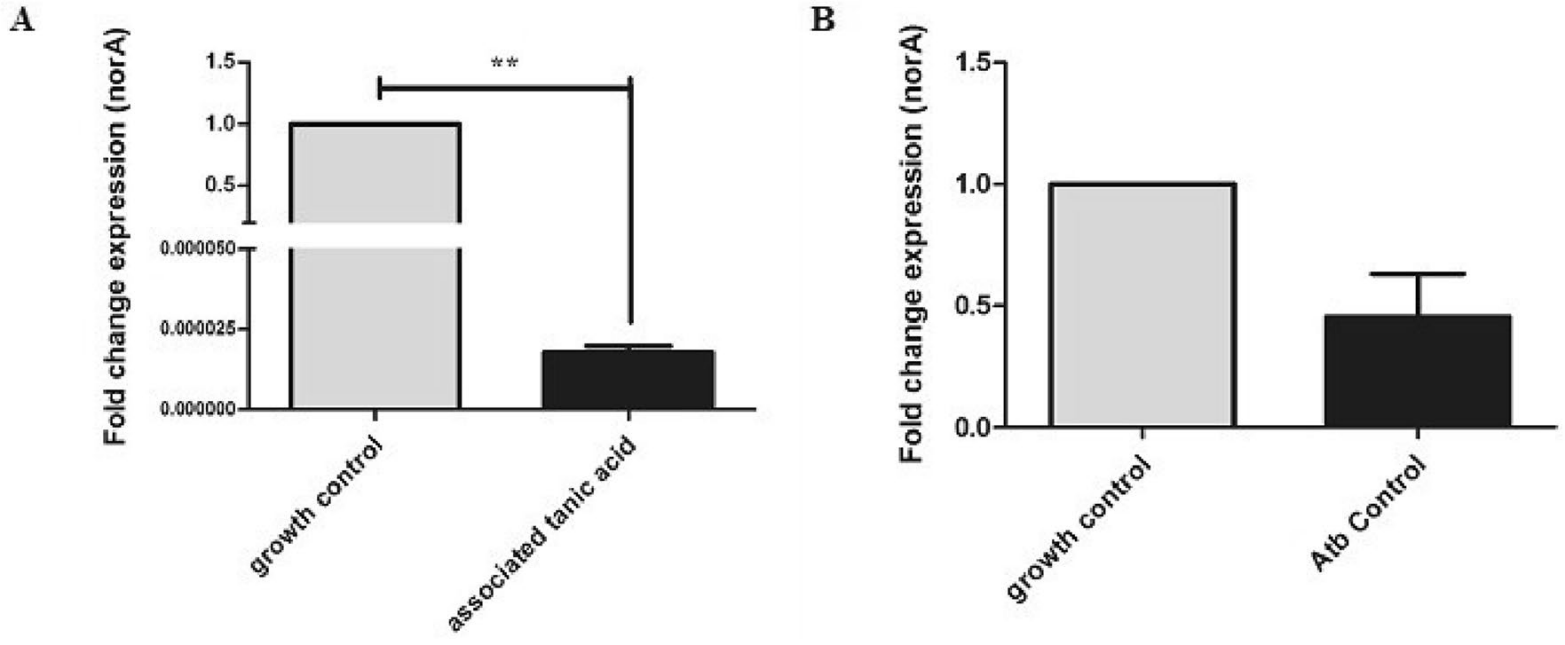
Relative *norA* gene expression in *S. aureus* 1199B in the presence of tannic acid associated with norfloxacin in comparison to the growth control: (**A**) comparison between growth control and association; (**B**) comparison between growth control and norfloxacin alone. **p < 0.01 vs control.

The relative *norA* gene expression in *S. aureus* strain was evaluated by RT-qPCR analysis in the presence of tannic acid. The results showed the *norA* gene had its expression significantly diminished in the presence of tannic acid (Fig. 2 and Table 2). In the presence of the other compounds, a reduction in *norA* gene expression was observed, was statistically significant.

**Table 2:** Relative expression of the *norA* gene in *S. aureus* strain in presence of tannic acid and antibiotic. **p < 0.01.

–	Control	Tannic acid + norfloxacin
CT mean—16S	31.907	22.528
CT mean—NorA	26.623	33.035
∆∆CT (Mean + SD)	1	0.0002 ± 0.000003**

### Determination of ethidium bromide fluorimetry in Gen5™ microplate reader

**Figure 3:**
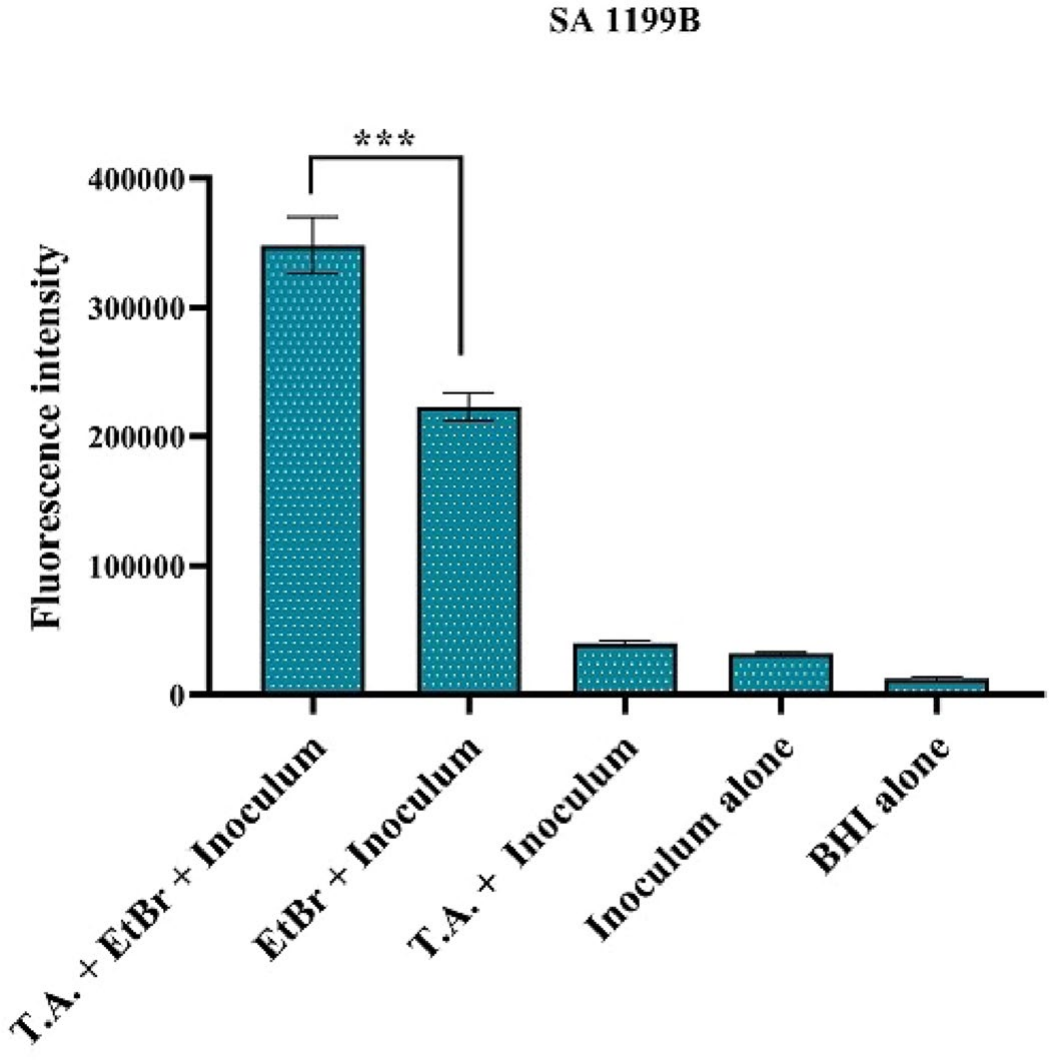
Evaluation of NorA efflux pump inhibition by the fluorescence emission. Method performed in Gen5™ Microplate Reader. T.A = Tannic acid; BHI = brain heart infusion agar; EtBr = ethidium bromide. ***p < 0.001 vs EtBr + inoculum.

In the accumulation of ethidium bromide in microdilution plate, a significant increase of ethidium bromide inside the cell was observed when treated with tannic acid. This increase was represented by the 34.9% increase in fluorescence intensity in the sample treated with tannic acid, indicating inhibition of the NorA efflux pump. Ethidium bromide with bacteria show lower values than those mentioned above. The treatments: tannic acid with bacteria, bacteria alone, and BHI alone did not show significant fluorescence (Fig. 3).

### Determination of ethidium bromide fluorimetry in UV transilluminator

**Figure 4:**
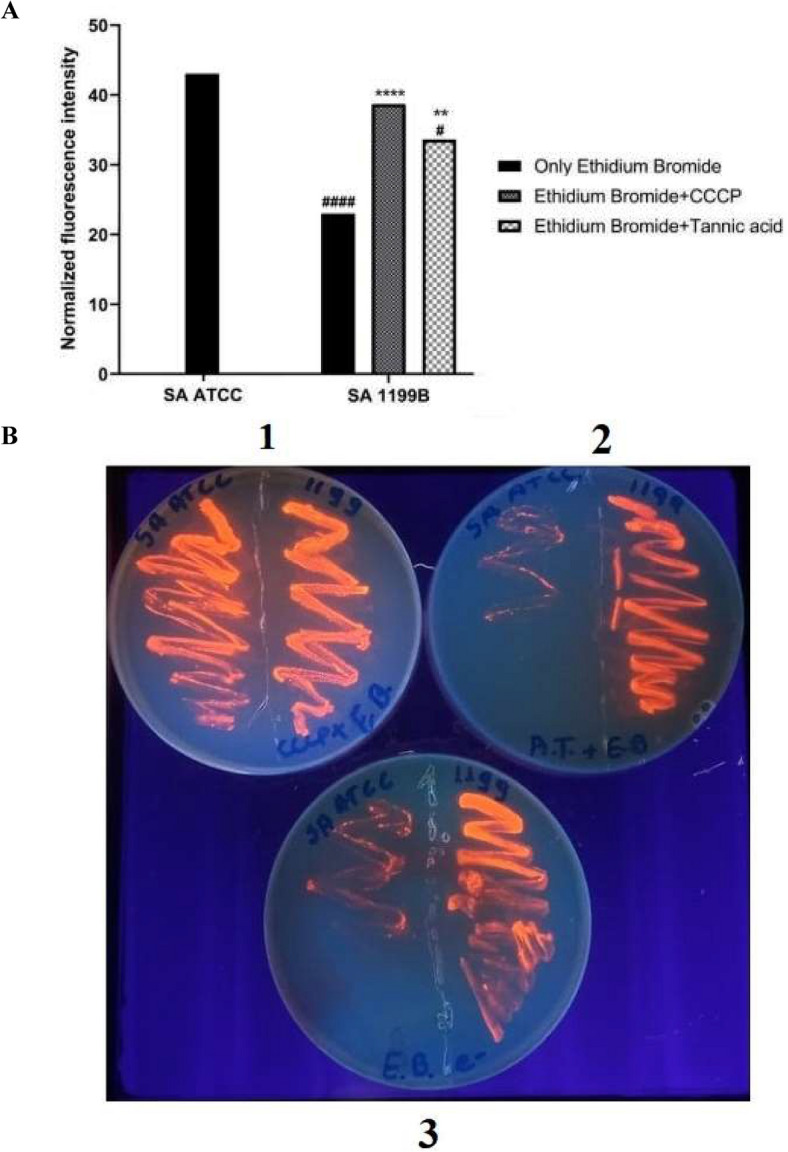
Evaluation of NorA efflux pump inhibition by the fluorescence emission. method performed in UV Transillumination. A = Quantitative analysis. B = Qualitative analysis; B1 = CCCP efflux pump + ethidium bromide; B2 = Tannic acid + ethidium bromide. B3 = ethidium bromide alone (negative control). SA = Staphylococcus aureus. ####p < 0.0001 vs SA ATCC; #p < 0.05 vs SA ATCC; ****p < 0.0001 vs SA 1199; **p < 0.01 vs SA 1199.

In plate visualization, the most intense glow was observed by ethidium bromide accumulation when treated with the efflux pump pattern inhibitor the CCCP. Similar results were observed with the presence of the tannic acid result, indicating that the tannic acid inhibits pump. As consequence the bromide accumulation give a more intense shine. This result is not observed in the strain when there are inhibitors not (Fig. 4A). In the strain without the *norA* pump gene (ATCC 25,923), maintenance of brightness was the same, both in the presence of tannic acid and in its absence. In the strain (1199) with baseline expression of the *norA* gene, greater brightness was observed when treated with tannic acid (Fig. 4B).

Fluorescence intensity was measured using Image J software (National Institute of Mental Health, Bethesda, MD, USA) from images captured with a 12 M digital camera. In the program, the normalized fluorescent intensity was evaluated by bacterial growth that was selected manually through the software, which calculated the area of a number of pixels in the chosen area (Area) and the brightness of the pixels (IntDen). The normalized fluorescent intensity was calculated by dividing the total brightness of the pixel area by the fluorescent area^23^.

## Checkerboard

A synergistic effect was also observed in the checkerboard method in the association of tannic acid + norfloxacin and tannic acid + EtBr, obtaining a FICI of 0.43 and 0.5 respectively. This reinforces the occurrence of potentiation of the effect of these substances and a putative inhibition of the efflux pump (Table 3).

**Table 3:** Synergistic effects of the Tannic acid with norfloxacin and/or EtBr, evaluated by the checkerboard method.

Bacteria	Compound	FIC	FICI	Outcome
SA-1199B	Tannic acid	0.12		
	Norfloxacin	0.31	0.43	Synergistic
	Tannic acid	0.25		
	EtBr	0.25	0.5	Synergistic

FICI = Fractional inhibitory concentration index (FIC index); EtBr = Ethidium bromide.

### Molecular docking

**Figure 5:**
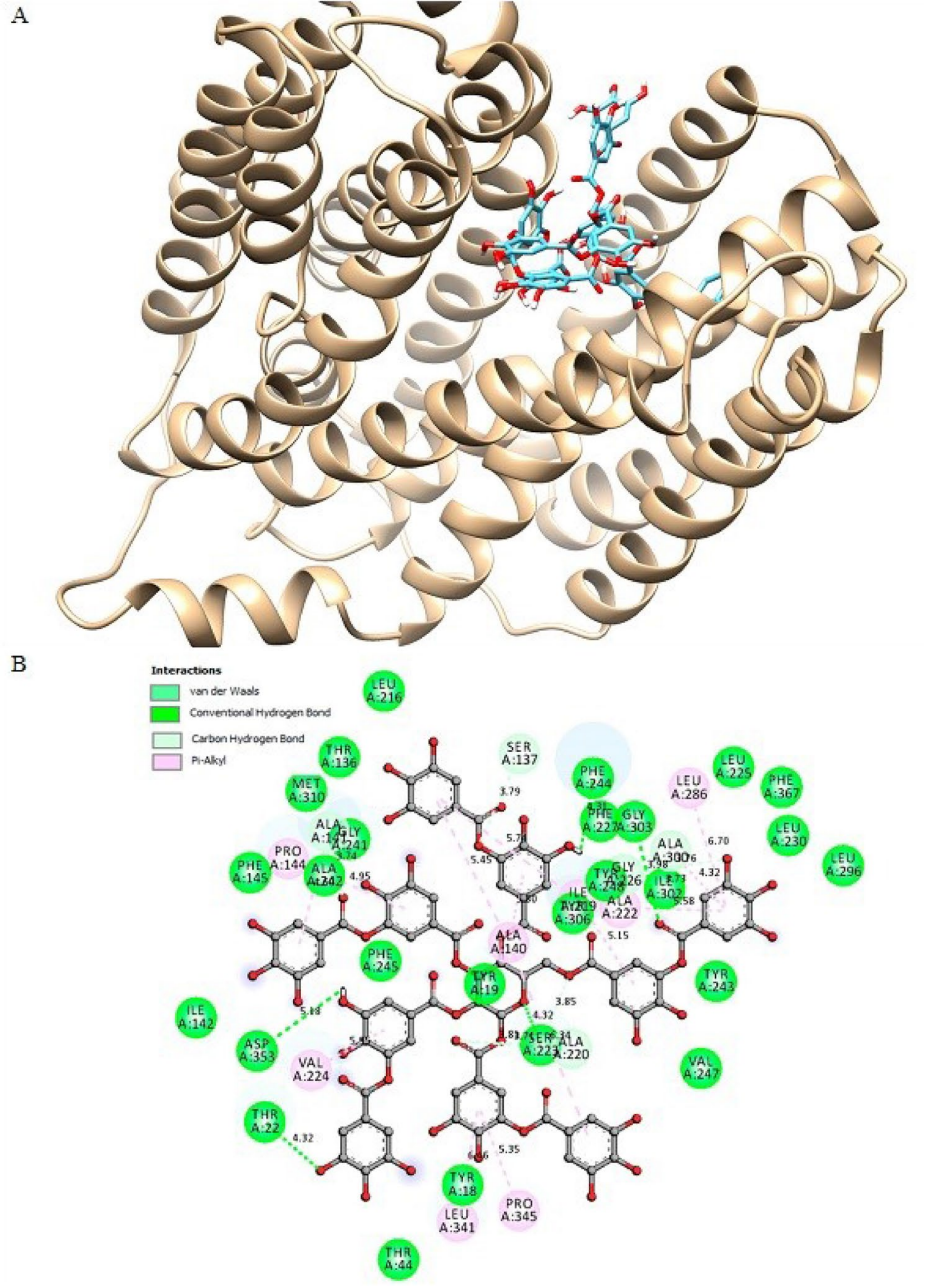
The visualization of the most energetically favorable binding of the NorA and tannic acid. (**A**) Predicted interaction between tannic acid visualized by Chimera. (**B**) representation showing 2D diagram of the NorA-Tannic acid molecular interactions.

The docking was carried out with SwissDock (http://www.swissdock.ch). Simulations were carried out for ligand-bound protein complexes to energy-minimize the docked complex and stabilize the same. Output clusters were obtained after each run and the results showed that cluster is having the best full interactions. The Docking studies revealed that tannic acid was recorded as the most favorable binding energy of −11.81 kcal/mol, has the best binding and stability (Fig. 5A). The results suggested that tannic acid present significant bond with NorA with different interactions type (Van der Waals, hydrogen bond, electrostatic effect and pi-alkyl interactions). The tannic acid made hydrogen bonds with NorA with amino acids PHE244 (2.32A), THR22 (2.09A), SER223 (1.87A), ASP353 (2.63A) and GLY303 (2.61A) and isoleucine 87, involved in stabilizations of complex. However, all key residues described above were found to interact with NorA with different interactions as shown in Fig. 5B.

## Discussion

As shown in Fig. 5, tannic acid showed high binding affinity for the NorA protein. Therefore, this shows great potential for inhibiting NorA efflux pumps, a result which corroborates with the study by Tintino et al*.*, where the tannic acid ability to reduce the minimum inhibitory concentration of both ethidium bromide and Norfloxacin, which constitutes efflux pump inhibition, is highlighted^19^.

According to Fig. 2, tannic acid showed an effective capacity to reduce NorA efflux pump genetic expression. Thus, a reduction in genetic expression levels was observed in the comparison between the bacterium alone and the bacterium associated with the antibiotic. Moreover, the antibiotic also showed an ability to reduce *norA* gene expression, although such expression was not significantly altered.

Increased resistance to fluoroquinolones, biocides and dyes has been associated with NorA mediated efflux via the increased expression of the *norA* gene. This increased expression can be either constitutive, through the acquisition of mutations in the *norA* promoter region, or inducible, through the action of regulatory proteins. In addition to alterations occurring in the *norA* promoter region, NorA production may also be regulated by several regulatory systems, albeit a clear regulatory pathway remains to be elucidated^24^.

According to Deng et al*.*, an increase in iron concentration in the medium decreased the expression of the *norA* gene^25^. In addition, a decrease in the *norA* expression regulator, fur, occurs with high iron concentration^25^. It is known in the literature that tannic acid is an iron chelator, thus when it is added to the medium the quantity of iron available to the bacteria decreases^26^. However, in the present study an expressive decrease in the NorA efflux pump gene occurred even with iron depletion, possibly due to chelation in the medium.

Therefore, this effect cannot be associated with the influence of iron. However, it is noteworthy that tannic acid has been long known for having an affinity for several proteins and NorA activation depends on a sequence of signaling proteins, it is therefore, possible that some of these pathways have been inhibited by tannic acid, resulting in the reduced gene expression observed^27^.

Due to the size of the tannic acid molecule, it is unlikely that it acted externally in the bacterial cell wall since the response presented by the molecule may be linked to membrane-associated signals or the pump itself, which when inhibited can act by negative feedback inhibiting its own expression^28^, Since it is already known that tannic acid acts upon eukaryotic cell signaling pathways, although in prokaryotes this has not yet been reported^29^, it is likely that the ArlRS membrane sensor is the membrane target for tannic acid.

*NorA* transcription has been shown to be regulated by the two-component ArlRS system and the global transcription regulator MgrA. An unknown 18 kDa protein binds multiple times to DNA sequences located upstream of the −35 region of the *norA* promoter^1^. ArlRS are important for regulating factors involved in adhesion, autolysis and proteolytic *S. aureus* activity^30^. The binding of this 18 kDa protein is modified by ArlS, which appears to function as the transmembrane sensor of a two component regulatory system, ArlR–ArlS. A mutation in *arlS* resulted in *norA* expression upregulation and also altered the *norA* growth-phase regulation^30^, Unknown mutations outside of the *norA* coding and promoter regions can also result in NorA overexpression and fluoroquinolone resistance^30^.

Although this was not evaluated in the present study, Tintino et al*.*, report a direct tannic acid activity over the NorA efflux pump in the literature such as in the study where the authors show the tannic acid capacity for inhibiting the NorA pump whereby tannic acid reduces the minimum inhibitory concentration of norfloxacin and ethidium bromide^18^. Therefore, the aforementioned study corroborates with the docking results obtained here. Considering that tannic acid showed strong binding to the NorA pump, this probably contributed to the direct pump inhibitory effect, as reported in the literature.

## Conclusion

The tannic acid ability to inhibit the NorA efflux pump may be associated with its ability to inhibit the genetic expression of this protein by acting on signaling pathways involving the ArlRS membrane sensor, as well as by acting directly with the NorA protein through direct interaction, as seen in the in silico approach and proven in the literature. The in silico approach has been of great importance as a versatile tool to develop fast and accurate target identification and prediction methods for drug discovery. The present study provided information on the NorA and tannic acid binding process. These results suggest hydrogen bonds and hydrophilic interactions between tannic acid and NorA may function as a negative modulator. In the present study binding analysis showed tannic acid and NorA have the most favorable binding energy which corroborates with other results.

## Data Availability

The datasets analysed during the current study are available in the PDB repository (PDB ID: 1PV4, 1PV7, 3WDO, 2GFP) and were acquired in the FASTA format through the NCBI resources.
